# Achylia Gastrica1Read at the thirty-ninth annual meeting of the Medical Association of Central New York, at Syracuse, October 16, 1906.

**Published:** 1907-06

**Authors:** I. H. Levy

**Affiliations:** Syracuse, N. Y.; Associate Professor of Clinical Medicine, College of Medicine, Syracuse University Physician to the Hospital of the Good Shepherd, Syracuse; 717 East Genesee Street


### Achylia Gastrica.[1]

By I. H. LEVY, M.D., Syracuse, N. Y.
Associate Professor of Clinical Medicine, College of Medicine, Syracuse University
Physician to the Hospital of the Good Shepherd, Syracuse.

THE physician who is in the habit of using the stomach tube for diagnostic purposes, frequently encounters a condition characterised by the absence of free hydrochloric acid, a low total acidity together with a complete or almost complete absence of the ferments. Einhorn, in 1892, applied the term achylia gastrica to the condition, and it is now generally so known. The disease may be either primary or secondary. The latter form is encountered in a variety of diseases. It is most frequently associated with carcinoma of the stomach. It is easy to understand why there should be no secretion when the entire mucous membrane is infiltrated or replaced by the new growth. It is not quite so plain why there should be an absence of gastric juice during the early stage of the growth, while it is still sharply localised to a small area. And it is still more of a mystery why it should be absent with carcinoma

---

1. Read at the thirty-ninth annual meeting of the Medical Association of Central New York, at Syracuse, October 16, 1906.

situated remote from the stomach. It may be found with cancer of any organ. During the past year I encountered it with carcinoma of the bladder some months before there was any evidence of bladder disease.

It is almost constantly found with pernicious anemia. The credit of having first called attention to the association of gastric atrophy with pernicious anemia, has usually been accorded to Fenwick, who, in 1877, reported several cases. As a matter of fact, as Welch points out, it was Austin Flint, who as early as 1860, directed attention to this association. The relation of the gastric atrophy to pernicious anemia has been the source of much speculation and debate. At first it was held that the blood condition was secondary to the atrophy. But gastric atrophy does not always lead to pernicious anemia. While on the other hand, pernicious anemia is encountered without any change in the stomach mucosa. Today it is generally believed that the two conditions do not stand in the relation of cause and effect. It is supposed that both are due to some common toxic cause. It may be encountered with other forms of anemia. I saw a case of splenic anemia in which repeated examinations of the stomach contents failed to reveal any free hydrochloric acid or the ferments. Atrophy of the gastric mucosa is not infrequently seen as the terminal stage of a chronic gastritis. It is found in the late stage of phthisis, sometimes with diabetes and with those disturbances of the heart, liver and kidneys, which lead to chronic gastritis.

Primary achylia gastrica, or achylia gastrica simplex, as Martius calls these cases, is not so serious a disease as the secondary form. There are no gross anatomic lesions to account for the achylia. That a true achylia is ever a purely functional disturbance is denied by some clinicians. However, the belief that it is so appears to be gaining ground. Many of these patients suffer from a variety of nervous symptoms, or at least possess a constitution predisposed to neurasthenia. The undisturbed general condition, despite the apparent severity of the malady, together with the fact that the secretion of the gastric juice sometimes returns after a prolonged absence, argues in favor of a nervous form. Einhorn reports a case in which the hydrochloric acid reappeared after an absence of five years. I was, recently, agreeably surprised to find free hydrochloric acid in the stomach contents of a very nervous woman in whom, previously, repeated examinations during a period of two years had failed to reveal any.

The failure of the stomach to secrete the gastric juice would, at first thought, lead to the inference that the digestive functions would seriously suffer. However, such is not necessarily the case. An achylia may exist for years without causing any symptoms and may then be discovered only accidentally. Hemmeter mentions a medical student who was totally ignorant of any abnormality, in whom the condition was discovered while making routine stomach analysis of the class. Einhorn reports one case that he had observed for four years and whose general condition, if anything, improved; and another in which he believes the condition had existed for over forty years. Ewald also treated a case in which the secretion was absent for two and-a-half years, yet the patient gained forty-two pounds. I have observed a number of cases who have more than held their own for over two years. The absence of serious disturbances, in these cases, proves that the stomach is not necessarily essential to life. Modern surgery has demonstrated that not only can life go on after its removal (as has been done for carcinoma) but that weight may be even gained afterward.

The intestine can perform not only its own functions but that of the stomach as well. The pancreatic juice with its triple ferment is capable of digesting the proteids, carbohydrates, and fats which make up our dietary. It is of the greatest importance, however, that the motor functions of the stomach should be unimpaired. If an atony or a stenosis be present the entrance of food into the intestines is interfered with, and digestion is hindered. The intestinal mucosa must also be intact. If there be an intestinal atrophy, nutrition must suffer severely. As a rule the motor function is retained in achylia not due to carcinoma obstructing the pyloric orifice.

The stomach not only empties itself thoroughly, but as a rule much quicker than normally. It is often necessary to aspirate within half an hour after the test breakfast to obtain sufficent contents for examination. The hypermotility is no doubt due to the absence of hydrochloric acid. Physiologists tell us that the closure of the pylorus is due to the irritating action of the hydrochloric acid on its mucosa, and it only opens when the alkaline intestinal secretion neutralises the acidity of the chyme in the upper portion of the duodenum. The absence of the hydrochloric acid would mean a continually relaxed pylorus through which the fool could readily escape. But sooner or later this vicarious action of the intestines begins to fail and the patient suffers more or less. He complains of a feeling of pressure or distress after eating. Severe pains are occasionally present, but as a rule they are absent. The appetite is usually poor; it may however be normal. Pain when present begins directly after eating and its severity frequently varies with the size and character of the ingested meal. Sometimes the pain is relieved by the taking of food, in this respect resembling cases of hyperchlorhydria, a fact to which Einhorn called attention. Frequently a variety of nervous symptoms are associated with the above. There may be headache, vertigo, disturbed sleep, a feeling of fatigue, and lack of ambition.

Occasionally the patients are aware of something wrong with their digestion although unable to accurately describe their symptoms. In fully half of the cases intestinal symptoms, sooner or later, make their appearance. Occasionally it is their presence that calls attention to the malady. Diarrhea is the most frequent bowel disturbance. The patients have from three to five movements daily, occasionally the bowels move several times in succession early in the morning before breakfast. Sometimes they move directly after a meal. The movements are preceded by rumbling and gurgling noises in the abdomen. There is no straining. Sharp attacks of colic are only rarely present. Examination of the stools, especially after the ingestion of raw or partly cooked meat, shows large masses of undigested meat, unchanged connective tissue, the residue of vegetable cells and little if any fat. The feces frequently have a very offensive odor. In some cases constipation is the rule or there may be alternating periods, of diarrhea and constipation.

When the diarrhea persists for any length of time, the general condition suffers, the patients lose in flesh, becoming weak and may even become cachectic. This diarrhea is one of the most characteristic symptoms of achylia gastrica. Occasionally it is the only symptom. There is nothing to call attention to the stomach as the offending organ. The patients go to the physician believing themselves suffering from some bowel disturbance. The importance of this form of dyspeptic diarrhea cannot be overstated. If properly interpreted it responds readily to treatment, while the ordinary treatment for diarrhea is entirely without effect. The reasons for the diarrhea are apparent. In the first place the food not being properly ground up in the stomach reaches the intestines in a coarse state, and accordingly increases its mechanical as well as its chemical labors. This extra work leads to inflammatory change in the intestinal mucosa eventually leading to atrophy. But long before the atrophy stage is reached diarrhea may make its appearance. The hydrochloric acid of the stomach besides having digestive function aids in the disinfection of the bowels. Experiments have shown, that while the jejunum is normally sterile, in cases of achylia it swarms with bacteria. Pawlow has shown that the entrance of the acidified chyme into the duodenum acts as a powerful stimulant of pancreatic secretion. The absence of the hydrochloric acid means a poor pancreatic secretion, with its attending imperfect intestinal digestion.

Cases of achylia may for convenience sake be divided into four classes. Class one, includes those cases in which the absence of the hydrochloric acid and ferments causes no symptoms. Naturally such cases do not come to the physicians for stomach trouble. The condition is only accidentally discovered. Class two, includes those cases suffering from stomach symptoms without any associated bowel disturbance. Class three, includes those suffering from stomach and intestinal symptoms, while class four, includes those presenting intestinal symptoms only. The following cases are illustrative of classes two, three, and four.

Case I. Mrs. C. aet. 40, usual diseases of childhood. Two years ago had an attack of neuritis. For some time past has had pain in the epigastic region. The pain is independent of the taking of food, yet a heavy meal aggravates it. Occasionally, it continues during the entire night. No nausea or vomiting, but belches and bloats. Constipated. Sleep disturbed. Very nervous. Examination of the stomach contents after the usual Ewald test breakfast shows the absence of free HCl. Total acidity, four. Filtered contents does not coagulate milk, nor acidulated will it digest a disk of albumen.

Case II. Mrs. C. aet. 44. After eating bloats and belches, appetite good, relishes everything. Headaches. Sleep disturbed, and very nervous. Constipated. Lungs and heart negative. Right kidney palpable. Examination shows the fasting stomach empty. After the usual test meal, the extracted contents shows the bread but slightly changed, and imbedded in the mucus. No free HCl. Total acidity, four.

Case III. Mrs. B. aet. 30. Perfectly well up to two years ago. Illness began with indefinite pains across the epigastrium. Appetite poor. Belches and bloats. Pains same day and night. Eating does not seem to affect them. Constipated. Fasting stomach empty. After test breakfast, the extracted contents showed the bread imbedded in a mass of mucus which passed through the tube with difficulty. Free HClO. Total acidity, four.

Case IV. Mrs. W., aet. 43. History negative, except she has always been constipated. For several years has been distressed after eating; nauseated at times, but did not vomit. About two years ago had an attack of diarrhea which lasted several months. She became weak and emaciated. She improved, but since then has had occasional attacks of diarrhea. She also suffers from very sharp pains in the epigastrium and abdomen. The pains come on about an hour after eating. Sometimes eating relieves them. She is nauseated. Vomits occasionally. Belches and bloats. Sleep is disturbed. Headache, dizzy, and very nervous. Examination shows the greater curvature three inches below the navel (gastroptosis). The right kidney is freely movable. After the test breakfast, the food is softened but otherwise unchanged. Free HClO. Total acidity, 8. Examination of the feces after a meat meal showed large masses of striated muscle fibre, much unchanged connective tissue, some vegetable cells, but little fat.

Case V., Mrs. S., aet. 30. History negative, except that she was always nervous. For the past two years has been troubled with attacks of diarrhea alternating with constipation. The diarrhea sometimes lasts but a few days, at other times it continues a month or more. She had four or five movements daily, usually after meals. The appetite was good, and her appearance that of a well nourished woman. There was nothing to call attention to the stomach as the offending organ. Examination of the stomach contents after the usual test breakfast failed to reveal any free HCl. The total acidity was 6. Examination of the feces showed large masses of undigested meat as in case four.

The diagnosis of achylia gastrica is very simple when the stomach tube is used. From the symptoms alone no positive diagnosis can be made, although when diarrhea is present it may be suspected. Sometimes the clinical picture resembles that of hyperchlorhydria. The absence of the free hydrochloric acid, and a total acidity not exceeding the acidity of the ingested meal makes the diagnosis. The ferments are usually absent although small quantities of pepsin may be shown to be present both in the stomach contents and in the urine by means of the more delicate tests used for this purpose. Much care however is needed to distinguish the primary from the secondary form.

It is not my intention to go into the details of treatment, but simply to touch on a few of the most important points. Of first importance is the diet. As the intestine is capable of digesting proteids, carbohydrates, and fats, a wide latitude in the choice of food may be allowed. Its preparation, on the other hand, is of the utmost importance. All meats must be thoroughly cooked so that the fibers easily separate. Connective tissue and tough fibrous meats must be avoided; likewise coarse fibrous vegetables. Thick vegetable purees are most suitable. The food must be thoroughly masticated. If the teeth are bad as they frequently are with digestive disturbances, the dentist's aid must be sought. Milk on account of the absence of the rennet, passes into the intestine unchanged, and is frequently not well borne.

Of drugs, hydrochloric acid and pepsin, especially the former, are of value. While it is not possible to introduce sufficient hydrochloric acid to equal the amount normally secreted by the stomach, good results are not infrequently obtained from small quantities. It is particularly valuable in cases complicated with diarrhea. In almost all of these cases improvement follows its administration. I am in the habit of following Ewald's plan of administering fifteen drops of the diluted hydrochloric acid one-half, one, and one and one-half hours after each meal. In some cases it also acts favorably on the nervous symptoms. Nux vomica, condurango, gentian, and the other bitter tonics are of use in combating the stomach distress. Lavage is only indicated when much mucus is secreted.

717 EAST GENESEE STREET.

